# Aortic dissection associated with cogans's syndrome: deleterious loss of vascular structural integrity is associated with GM-CSF overstimulation in macrophages and smooth muscle cells

**DOI:** 10.1186/1749-8090-5-66

**Published:** 2010-08-21

**Authors:** Gabriele Weissen-Plenz, Ömer Sezer, Christian Vahlhaus, Horst Robenek, Andreas Hoffmeier, Tonny DT Tjan, Hans H Scheld, Jürgen R Sindermann

**Affiliations:** 1 Department of Thoracic and Cardiovascular Surgery, University Hospital of Muenster, Albert-Schweitzer-Strasse 33, 48149 Muenster, Germany; 2 Leibniz Institute for Arteriosclerosis Research, Domagkstrasse 3, 48149 Muenster, Germany; 3 Department of Cardiology and Angiology, University Hospital of Muenster, Albert-Schweitzer-Strasse 33, 48149 Muenster, Germany

## Abstract

**Background:**

Cogan's syndrome is a rare disorder of unknown origin characterized by inflammatory ocular disease and vestibuloauditory symptoms. Systemic vasculitis is found in about 10% of cases.

**Case presentation:**

A 46-year-old female with Cogans's syndrome and a history of arterial hypertension presented with severe chest pain caused by an aneurysm of the ascending aorta with a dissection membrane located a few centimeters distal from the aortic root. After surgery, histopathological analysis revealed that vascular matrix integrity and expression of the major matrix molecules was characterized by elastolysis and collagenolysis and thus a dramatic loss of structural integrity. Remarkably, exceeding matrix deterioration was associated with massively increased levels of granulocyte macrophage colony stimulating factor (GM-CSF).

**Conclusion:**

Our data suggest that the persistently increased secretion of the inflammatory mediator GM-CSF by resident inflammatory cells but also by SMC may be the trigger of aortic wall structural deterioration.

## Background

Cogan's syndrome is a rare disorder of unknown origin characterized by inflammatory ocular disease and vestibuloauditory symptoms [1,2]. Major clinical features are interstitial keratitis and vestibuloauditory dysfunction. The variety of systemic manifestations includes fever, splenomegaly, lymphadenopathy, and musculoskeletal complaints. Systemic vasculitis is found in about 10% of cases and may involve the large vessels, appearing as Takayasu-like vasculitis with affection of the aortic valve but also the coronary arteries and the small kidney vasculature. Aortic aneurysms due to aortitis often refrain from being recognized in Cogan's syndrome, and are potentially fatal, with two of eight reported cases dying from aneurysm/arterial rupture [3,4]. To the best of our knowledge, Cogan's syndrome complicated by aortic dissection as mirrored by the present paper has not been described in detail yet.

## Case presentation

**Figure 1:**
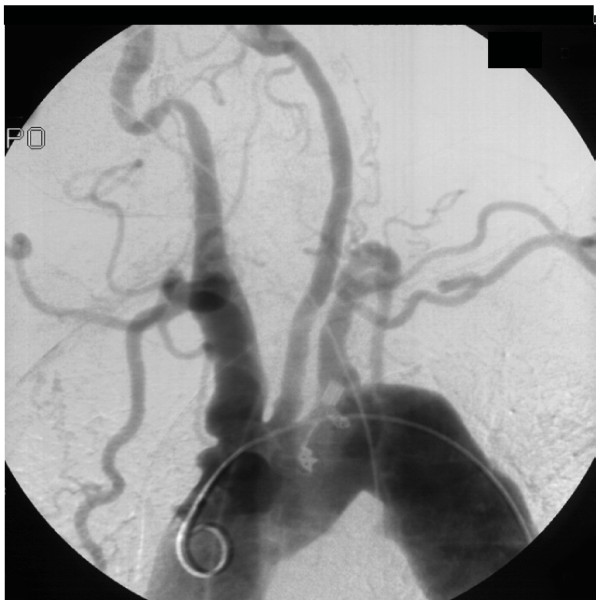
**Angiogram showing alternating dilatation and stenosis with irregularities of the aortic arch**. Both subclavian and vertebral arteries are occluded. The truncus bracheocephalicus is aneurysmatically dilated.

**Figure 2:**
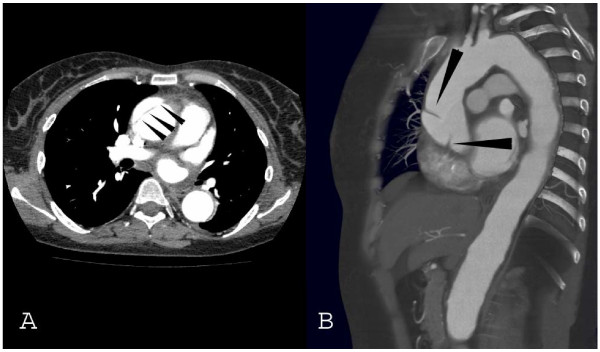
**Transverse (A) and sagittal (B) CT views of the aorta**. The dissection (arrows) is circumferential and begins distally to the coronary ostiae. arrows: dissection membrane

A 46-year-old female with Cogans's syndrome and a history of arterial hypertension was admitted with severe chest pain. Briefly, physical examination revealed the absence of radial pulses and no measurable blood pressure of the upper extremities. Aortic angiography revealed an aortic arch syndrome with occlusion of both subclavian and vertrebral arteries (Fig. 1). Ultrasound showed a fusiform abdominal aortic aneurysm and axial computed tomography demonstrated an aneurysm of the ascending aorta with a dissection membrane located a few centimeters distal from the aortic root (Fig. 2). At surgery the ascending aorta showed severe signs of inflammation comprising reddish alterations of the aortic walls and a pattern of thickened as well as thinned wall areas of the aorta and adjacent vessels. The ascending aorta revealed a circumferential dissection distal from the coronary ostia associated with an overload of pastuous material. The patient underwent a replacement of the ascending aorta by a 28 mm Vacscutek^® ^tube graft. After uneventful recovery the patient was discharged on postoperative day 17 with minimal and irrelevant neurological deficits.

**Figure 3:**
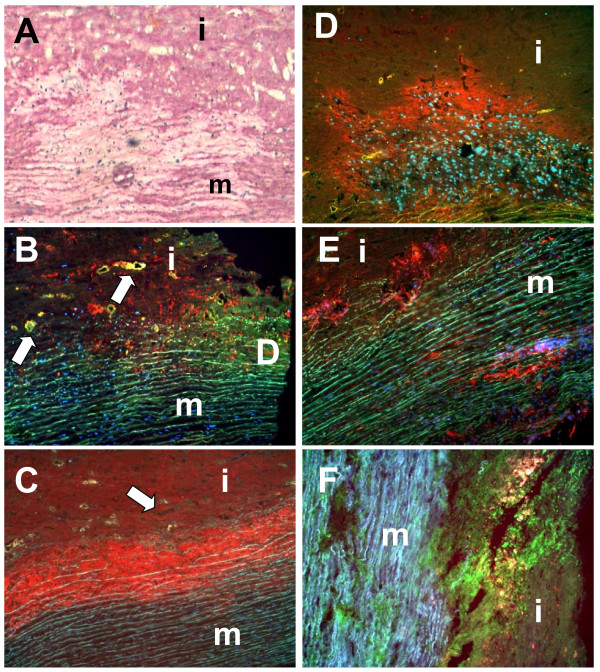
**Correlation between inflammatory infiltration and collagen degradation**. Picro Siriusred staining (panel A: collagen deposition = red) showing areas with massive collagen degradation. Immunohistochemistry for CD68 (panel B) and 27E10 (panel C) demonstrated accumulation of infiltrating cells particularly at the intima-media border (arrows indicate areas with profound vascularization). MMP1 (panel D) and MMP9 (panel E) were strongly expressed at the intima-media border and in deeper areas of the media. Interestingly, MMP2 expression was only very low (not shown). In situ zymography (panel F: collagenolysis) showed massive collagenolytic activity at the intima-media border and in deeper areas of the media. (m: media; i: intima; original magnification: ×100)

**Figure 4:**
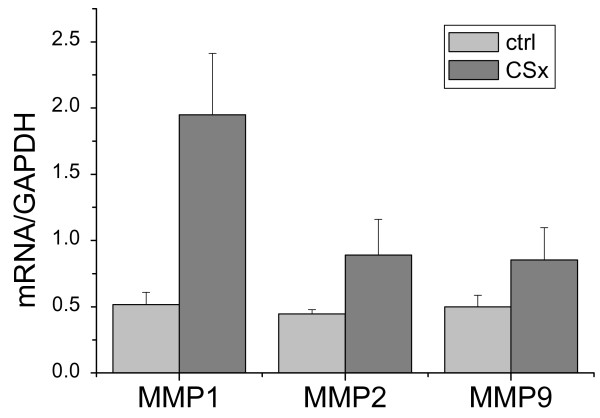
**Expression of collagenolytic MMPs as demonstrated by RT-PCR analyses**. Our data show a strong upregulation of the mRNA expression of collagenase (MMP1) and both gelatinases (MMP2 and MMP9) (CSx) compared with controls (ctrl).

Histological examination of the resected tissue specimen (Fig. 3) demonstrated an occult inflammatory process with massive inflammatory infiltration and loss of smooth muscle cells particularly at the intima-media border. Elastic lamellae and collagenous matrix were degraded (Fig. 3A). Foci of matrix deterioration in deeper medial areas collocated with clusters of inflammatory cells (Fig. 3B and 3C). MMP1 (Fig. 3D) and MMP9 (Fig. 3E) were strongly expressed at the intima-media border and in deeper areas of the media. Interestingly, MMP2 expression was only very low (not shown). Expression of collagenolytic MMPs was also studied by RT-PCR analyses (Fig. 4). Our data show a strong upregulation of the mRNA expression of collagenase (MMP1) and both gelatinases (MMP2 and MMP9) compared with controls. Zymography (Fig. 3F) revealed massive matrixmetalloproteinase activity at the intima-media border (rich in activated 27F10-positive inflammatory cells) and in association with clusters of CD68-positive macrophages in deeper medial areas. mRNA-expression analysis revealed a distinct increase in the expression profiles of the major vascular collagens (collagen type I, III, and VIII), which were expressed about 1.5 - 2-fold higher than in healthy control tissues. Similarly, tropoelastin was found to be 2-fold increased (data not shown).

**Figure 5:**
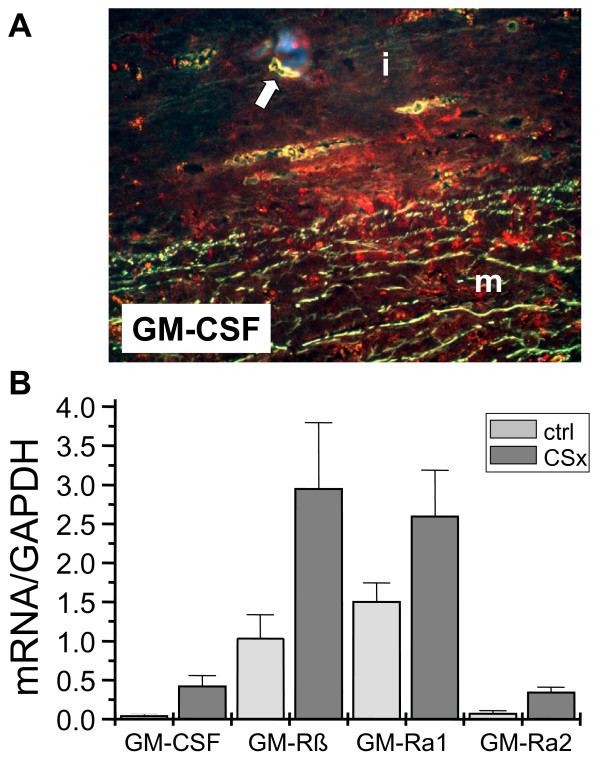
**Expression of the GM-CSF system**. A: Our data show a strong expression of GM-CSF at the intima-media border (arrows indicate areas with profound vascularization). B: RT-analyses revealed that not only GM-CSF but also its receptor subunits - the common receptor untit GM-CSF-receptor ß (GM-Rß) and both (-Subunits (GM-Ra1 and GM-Ra2) - are upregulated in CSx compared with controls (ctrl).

In areas of exceeding matrix deterioration the level of the granulocyte macrophage colony stimulating factor (GM-CSF), was massively increased. The gross of GM-CSF was collocated with macrophages, but also with neighboring SMC. In comparison with normal controls (aortic specimens taken during ACB surgery, N = 10) overall mRNA expression of GM-CSF and its receptor subunits was distinctively elevated (N = 3) (Fig. 5).

## Conclusion

Taken together, our data on vascular matrix integrity and expression of the major matrix molecules indicate that although both collagen and elastin production was stimulated in SMC, the even greater increase in MMP activity in both inflammatory cells and SMC resulted in exceeding elastolysis and collagenolysis and thus a dramatic loss of structural integrity.

Interestingly, in areas of exceeding matrix deterioration the level of GM-CSF, a proinflammatory mediator and important regulator of the vascular collagen and elastin metabolism [5,6], was massively increased.

In summary, our data suggest that the persistently increased secretion of the inflammatory mediator GM-CSF by resident inflammatory cells but also by SMC may be the trigger of aortic wall structural deterioration. GM-CSF is a regulator of vascular collagen and elastin metabolism. In mice with genetically modulated GM-CSF-expression adverse remodeling of the vascular extracellular matrix was found [5,6]. Therefore, overstimulation of the GM-CSF-system as found in our patient may present the underlying trigger of adverse extracellular matrix remodeling, loss of functional texture (in the areas of persistent inflammation) and thus contribute to the destabilization of the aortic wall finally leading to aortic dissection.

Cogan's syndrome complicated by aortic dissection has not been described in detail yet. The herein presented case of a 46-year old female suggests that overstimulation of the GM-CSF-system may function as an underlying trigger of adverse extracellular matrix remodeling and loss of functional texture. These features may contribute to the destabilization of the aortic wall which may provide a mechanism leading to aortic dissection in a number of vascular diseases.

## Competing interests

The authors declare that they have no competing interests.

## Authors' contributions

All authors have read and approved the final manuscript. Contributions were as follows: GW-P: PhD, Main author, histologic studies, contribution to arrangement of the manuscript; ÖS: MD, assembly of clinical data, contribution to arrangement of the manuscript; CV: MD, contribution to clinical vascular studies; HR: PhD: Head of laboratory where histologic studies were performed; AH: MD, cardiac surgeon, contribution to surgical procedure; TDTT: MD, cardiac surgeon, contribution to surgical procedure; HHS: MD, head of the Departement of Thoracic and Cardiovascular Surgery; JRS: MD, cardiologist, contribution to arrangement of the manuscript.

## Consent

The study was approved by the local Review board of the University of Muenster. Written informed consent was obtained from the patient for publication of this case report and accompanying images. A copy of the written consent is available for review by the Editor-in-Chief of this journal.
